# Correlates of quality of life among persons living with tuberculosis: A cross-sectional study

**DOI:** 10.1371/journal.pone.0277192

**Published:** 2022-11-04

**Authors:** Eric Tornu, Louisa Quarcoopome

**Affiliations:** 1 Department of Adult Health, School of Nursing and Midwifery, College of Health Sciences, University of Ghana, Legon, Accra, Ghana; 2 Department of Internal Medicine and Therapeutics, Korle-Bu Teaching Hospital, Korle-Bu, Accra, Ghana; PLOS (Public Library of Science), UNITED KINGDOM

## Abstract

**Introduction:**

The correlates of quality of life originating from the demographic characteristics, comorbidities and sources of social support among persons living with tuberculosis remain underreported. The aim of this study was to examine the correlates of quality of life among persons living with tuberculosis within Greater Accra, Ghana.

**Methods:**

A cross-sectional survey design was used to assess the correlates of quality of life among 250 randomly sampled persons living with tuberculosis. Quality of life was assessed with the Quality of Life Brief Version (WHOQOL-BREF) questionnaire and correlates were derived using Spearman *rho* correlations. Chi-square analyses assessed associations among respondent characteristics.

**Results:**

All four quality of life domains (physical, psychological, social relationship and environmental) of persons living with tuberculosis were positively correlated. Furthermore, receiving social support from family or friends correlated positively with respondents’ quality of life. Human Immunodeficiency Virus (HIV) infection and receiving social support mainly from work colleagues or religious institutions correlated negatively with respondents’ quality of life domains. Other correlates of quality of life included age, pleuritis with pleural effusion, number of dependants and distance to tuberculosis treatment centres. Social support from family and friends corresponded with better quality of life among persons living with tuberculosis.

**Conclusion:**

The quality of life domains of persons living with tuberculosis are interrelated and can be enhanced by social support. Healthcare providers should involve the significant others of persons living with tuberculosis, human immunodeficiency virus and pleuritis with pleural effusion in their care to promote patients’ quality of life.

## Introduction

Tuberculosis (TB) is a deadly airborne disease that is currently the second leading infectious disease killer after being overtaken by the coronavirus disease in 2020 [1]. Out of the over 10 million people who fell ill with TB globally in 2020, about 2.5 million were in Africa, of which over half a million were living with human immunodeficiency virus infection (HIV) [2, 3]. Immunocompromised persons such as persons living with HIV or diabetes mellitus are more likely to develop TB disease and face a higher risk of death on account of TB [4, 5]. Of the 1.5 million persons that died worldwide on account of TB in the year 2020, about 214,000 were HIV positive of which 79% were living in Africa [2, 3]. Even though TB predominantly affects the lungs, the inflammatory process initiated by TB infection may lead to pleuritis which is an inflammation of the pleural lining around the lungs [6]. It is estimated that about 3% to 25% of persons living with TB (PLWTB) develop pleuritis which results in an accumulation of lymphocytic fluid within the pleural spaces termed pleuritis with pleural effusion [7].

The incidences and mortality associated with TB in Ghana have declined steadily over the past two decades with a current incidence of 44,000 and mortality of 14,900 (33% among persons with HIV) [8]. The Ghana National TB control Program’s implementation of the Direct Observed Treatment Short-course (DOTS) has made antituberculosis chemotherapy accessible nationwide and contributed to the reduction in TB-related illnesses and deaths [9]. The PLWTB undergo antituberculosis chemotherapy in DOTS or TB treatment centres for a minimum of 6 months with an extendable duration of treatment to ensure complete cure [10]. However, much of the TB control efforts have focused on preventing, diagnosing and treating TB in the population [11]. The impact of TB on the health-related quality of life (QOL) of PLWTB requires similar attention and prioritization.

The QOL of individuals comprises of their perception regarding their position in life within the context of the culture and value system within which they live and based on their goals, standards, concerns and expectations [12]. The QOL embodies a person’s overall wellbeing and may be related to the physical, psychological, social relationship and environmental domains of their lives. It is important to consider the QOL of PLWTB because there is evidence that TB affects the physical, psychological, social relationship and environmental wellbeing of PLWTB despite the initiation of TB treatment [13–16]. For instance, in the Physical QOL domain, PLWTB may still experience bodily pains, sleep disturbances or low energy levels. In the psychological QOL domain, PLWTB may experience stigmatization which could be a barrier to their care as well as impaired ability to concentrate and enjoy life [14]. The environmental QOL domain of PLWTB considers their satisfaction with their living spaces, access to health services and transportation [17]. Tuberculosis infection may also affect the social relationship QOL domain of PLWTB where their satisfaction with their personal relationships, sex life and extent of social support received from friends may be affected upon diagnosis with TB [18, 19].

Social support refers to the care and assistance that is available to an individual from others in their social network [20, 21]. Social support for PLWTB may be in the form of providing them with health information, emotional support (care to deal with psychological challenges), companionship support (care which engenders a feeling of companionship) and material support (provision of commodities and finances) [20]. The providers of social support for ill persons such as PLWTB include their family, friends, community members, faith-based or religious organizations and healthcare providers [22, 23]. In some cases, PLWTB are the breadwinners of their social network thus must provide for the sustenance of their social support providers (such as family members) who double as their dependants. Even though this situation can be a strain on convalescent PLWTB, the availability of social support remains a valuable factor in the recovery and QOL of PLWTB [19].

Beyond social support, results from existing studies indicate that the factors associated with the QOL of PLWTB include their age, sex, type of TB, comorbidities (such as HIV infection), adherence and duration of TB disease [17, 24–27]. Despite the existence of these studies globally, there is a paucity of research that have examined the correlates of QOL originating from the demographic characteristics, comorbidities and sources of social support among PLWTB within a resource-limited setting like Ghana. Resource-limited settings particularly in Sub-Saharan Africa lack the requisite resources to completely tackle the high incidences and deaths associated with TB [5]. There is therefore the need to identify the relevant correlates of QOL among PLWTB in such settings to efficiently target available resources and improve patients’ health outcomes. The findings of this study can provide insight to healthcare professionals, researchers and policymakers regarding the correlates of QOL to consider (and possibly prioritise) in their care, research or policies related to the QOL of PLWTB. This study thus examined the correlates of QOL among PLWTB in Greater Accra, Ghana.

## Materials and methods

### Study design and setting

This descriptive cross-sectional survey examined the QOL of PLWTB from March 2021 to October 2021. The study included PLWTB who were receiving antituberculosis treatment in the DOTS or TB treatment centres of four (4) public health facilities in Greater Accra, Ghana. They comprised two municipal hospitals, a general hospital and a teaching hospital. Whereas the municipal hospitals predominantly provided primary healthcare to patients, the general hospital offered in-patient and out-patient care health services to PLWTB-HIV. The teaching hospital provides in and outpatient health services as well as specialized thoracic surgeries for PLWTB. The Greater Accra Region, Ghana’s mainly urban capital, has one of the country’s greatest concentrations of PLWTB thus was selected as the regional setting of the study. Ghana is a low-to-middle income West African country with about 9000 accredited DOTS health facilities nationwide which provide health care to its population of 30 million citizens [28].

### Population

The study included PLWTB in Greater Accra, Ghana, who were l5 years or older, had been diagnosed with the TB disease and were being managed with antituberculosis medication through DOTS. The PLWTB were included in the study irrespective of their site of TB infection (pulmonary or extrapulmonary TB), comorbidity status (TB with HIV, diabetes mellitus or pleuritis with pleural effusion) or duration of TB treatment. The study excluded PLWTB who had been diagnosed with drug-resistant TB. The exclusion was on account of the difference in TB treatment regimens for persons living with drug-sensitive and drug-resistant TB. Respondents’ sample size was determined prior to the study using G*Power software (version 3.1.9.7). We assumed an effect size of 0.5, a power of 0.8 and an alpha value of 0.05 (two-tailed) which yielded a minimum of 128 respondents [29]. Nonetheless, 250 PLWTB were sampled to account for non-response and ensure adequate respondent sampling.

### Ethical consideration

The Ghana Health Service Ethics Review Committee (GHS-ERC 027/01/21) granted ethical clearance and the administration of the four (4) health institutions provided permission for the study. The study adhered to the ethics of voluntary involvement, beneficence and non-maleficence, privacy and secrecy by only enrolling informed and consenting participants who had signed written informed consent forms. Respondents were free to leave the research at any moment and without giving any reason.

### The instrument

The physical, psychological, social relationship and environmental QOL of PLWTB were assessed using the English language version of the World Health Organization’s Quality of Life Brief Version (WHOQOL-BREF) questionnaire [30]. The 26-item WHOQOL-BREF measure is divided into four domains: physical (7 questions), psychological (6 questions), social relationship (3 questions) and environmental (8 questions). Two (2) generic questions assessed respondents’ overall QOL and health satisfaction. The respondents’ answers were graded on a 5-point Likert scale. Each QOL domain’s raw scores were translated into a range of zero (0) to 100. Thus, higher QOL domain scores represented better QOL in each domain according to the WHOQOL-BREF developers’ instructions [30]. A QOL domain score below 50% was considered as low QOL in that domain. The WHOQOL-BREF is a reliable and valid instrument that has been used globally to measure the QOL of PLWTB thus was considered suitable for the current study [13, 14, 17]. The instrument was pre-tested among 50 PLWTB in Greater Accra, Ghana to ensure it was valid and reliable for this study. The pre-test respondents who met the study’s inclusion criteria were randomly selected from the study setting. The pre-test data was separated from the main study data. An overall Cronbach’s alpha value of 0.95 was reported in this investigation, thus the WHOQOL-BREF was determined to have adequate reliability [31]. No changes were made to the 26 items of the QOL domains. Apart from the QOL domains, the WHOQOL-BREF elicited data on respondents’ age, comorbidities (HIV infection, pleuritis with pleural effusion, diabetes mellitus), duration of TB treatment, number of dependants and distance to their TB treatment centre. The final part of the WHOQOL-BREF assessed respondents’ main source of social support in dealing with the impact of TB on their QOL.

### Data collection procedure

With the help of professional nurses caring for PLWTB at the four (4) health institutions, the researchers engaged and randomly recruited potential respondents. The nurses described the study’s objective and procedures to each potential respondent and the researchers addressed their questions regarding the study. Potential respondents were contacted individually at the TB treatment centre on their hospital visit days after they had received treatment. Respondents who volunteered were given informed consent forms to fill out. Six (6) of the 256 PLWTB who were approached declined to participate in the study because they were not interested. The 250 willing participants (97.7%) signed the informed consent form and completed hard copies of the WHOQOL-BREF. The researcher read the questionnaire out loud to nine (9) non-literate respondents and recorded their replies on their questionnaires. The WHOQOL-BREF was completed by respondents in less than 60 minutes and collected by the researcher for data analysis.

### Data analysis

The data was analyzed using the Windows version of the Statistical Package for Social Sciences (SPSS) (version 23, IBM Inc). Descriptive statistics, including frequencies and percentages, were used to report respondents’ demographic characteristics, their QOL and their main source of social support against the impact of TB. Data were summarized using frequency tables. Spearman’s *rho* correlation analysis was used to assess the magnitude and direction of the relationship between respondents’ characteristics (age, HIV infection comorbidity, pleuritis with pleural effusion comorbidities, duration on TB treatment, number of dependants and distance to TB centre), their QOL domain scores (physical, psychological, social relationship and environmental domains) and their main source of social support against the impact of TB (family, friends, work colleagues or religious institution). Statistically significant (*p* < .05) Kolmogorov-Smirnov tests of normality indicated that the continuous variables (age, distance to TB treatment centre, duration of TB treatment, number of dependants and the QOL domains) were not normally distributed thus Spearman correlation was considered to be suitable [32]. The five (5) dichotomous variables included in the correlation matrix were analysed after dummy coding as follows; HIV infection (0 = no HIV infection, 1 = HIV infection), pleuritis with pleural effusion (0 = no pleuritis with pleural effusion, 1 = has pleuritis with pleural effusion), support from family (0 = not support from family, 1 = support from family), support from friends (0 = not support from friends, 1 = support from friends), support from work colleagues (0 = not support from work colleagues, 1 = support from work colleagues) and support from religious institutions (0 = not support from religious institutions, 1 = support from religious institution). Listwise deletion of cases was employed to ensure a constant sample size of 239 for all correlation analyses. Chi-square analyses were conducted to identify the association among respondents’ characteristics. A *p* < 0.05 level of significance was used for all statistical tests.

## Results

### Respondents’ demographic characteristics

Overall, 250 respondents participated in the study. As presented in Table 1, the respondents’ mean age was 38.81 (standard deviation = 14.26) years and ranged from 16 to 88 years. Most of the respondents were female (150, 60%), however, 16% (40) of all respondents had been diagnosed with co-morbidities including HIV (11, 4.4%) and pleuritis with pleural effusion (31, 12.4%). None of the PLWTB sampled had been diagnosed with diabetes mellitus. Family members (137, 54.8%) were the main source of social support for the majority of the respondents in dealing with the impact of TB.

**Table 1:** Demographic characteristics of people living with Tuberculosis (*N* = 250).

Characteristic		Frequency	Percentage
Age	Mean (*SD*): 38.81 (14.26) years	250	
Sex	Female	100	40
	Male	150	60
Educational level	No formal education	35	14
	Primary	78	31.2
	Secondary	96	38.4
	Tertiary	41	16.4
Employment status	Student	42	16.8
	Unemployed	50	20
	Employed	158	63.2
Phase of Treatment	Intensive	161	64.4
	Continuous	89	35.6
Co-morbidity	Absent	210	84
	Present	40	16
Category of TB case	New case	247	98.8
	Retreatment	3	1.2
Site of TB	Pulmonary	182	72.8
	Extrapulmonary	68	27.2
HIV status	Negative	239	95.6
	Positive	11	4.4
Pleuritis with pleural effusion	Absent	219	87.6
	Present	31	12.4
Diabetes Mellitus	Absent	250	100
	Present	0	0
Main source of support in dealing with the impact of Tuberculosis	Family	137	54.8
	Friends	57	22.8
	Work colleagues	22	8.8
	Religious institutions	34	13.6

HIV = Human Immunodeficiency Virus

### Correlation between respondents’ characteristics, source of social support and quality of life scores

There was a statistically significant positive correlation between respondents’ Physical QOL and their Psychological QOL (*r* = .80, *p* < .001), Social relationship QOL (*r* = .68, *p* < .001) and Environmental QOL (*r* = .43, *p* < .001) as well as receiving support from friends *(r* = .18, *p* = .005). However, a negative correlation was observed between Physical QOL and age *(r* = -.25, *p* < .001), having HIV infection *(r* = -.18, *p* = .005), having pleural effusion *(r* = -.21, *p* = .009), number of dependants *(r* = -.16, *p* = .016) and receiving support mainly from religious institutions *(r* = -.22, *p* = .001).

Psychological QOL was negatively correlated with HIV infection *(r* = -.22, *p* = .001), distance to TB centre *(r* = -.14, *p* = .026), receiving support mainly from work colleagues *(r* = -.14, *p* = .031) and religious institutions *(r* = -.31, *p* < .001). Nonetheless, Psychological QOL was positively correlated with Social relationship QOL (*r* = .74, *p* < .001) and Environmental QOL (*r* = .69, *p* < .001) as well as receiving support mainly from family (*r* = .18, *p* = .005) and friends (*r* = .14, *p* = .028).

A negative correlation was observed between respondents’ Social relationship QOL and HIV infection (*r* = -.27, *p* < .001), pleuritis with pleural effusion (*r* = -.13, *p* = .044), distance to TB centre (*r* = -.13, *p* = .045) and receiving support mainly from religious institutions (*r* = -.23, *p* < .001). Inversely, Social relationship QOL was positively correlated with Environmental QOL (*r* = .52, *p* < .001).

Environmental QOL was negatively correlated with HIV infection *(r* = -.15, *p* = .021), receiving support mainly from work colleagues *(r* = -.14, *p* = .035) and religious institutions *(r* = -.34, *p* < .001). Nonetheless, high Environmental QOL scores corresponded with older respondent age *(r* = .14, *p* = .035) and receiving support mainly from family (*r* = .35, *p* < .001). Chi-square analysis revealed significant associations between pleuritis with pleural effusion comorbidity and support originating mainly from friends [*X* ^2^ (1, *N* = 250) = 4.67, *p* = .037].

Further analysis revealed that older age corresponded with shorter duration of TB treatment (*r* = -.21, *p* = .001) and receiving less support from work colleagues (*r* = -.18, *p* = .004). However, older age correlated with a higher number of dependants (*r* = .54, *p* < .001) and longer distances to the TB centre (*r* = .26, *p* < .001). A positive correlation was also observed between HIV infection and number of dependants (*r* = .23, *p* < .001).

Having pleuritis with pleural effusion correlated negatively with respondents’ duration on TB treatment (*r* = -.15, *p* = .019) and receiving support mainly from friends (*r* = -.15, *p* = .019). Inversely, pleuritis with pleural effusion correlated positively with distance to TB centre (*r* = .23, *p* < .001) and receiving support mainly from religious institutions (*r* = .16, *p* = .011). Respondents’ duration on TB treatment correlated negatively with their number of dependants (*r* = -.16, *p* = .016) and distance to TB centre (*r* = -.26, *p* < .001). Table 2 summarises the correlations observed between respondents’ characteristics (age, HIV infection status, pleuritis with pleural effusion status, duration of TB treatment and distance to TB treatment centre), source of social support (support from family, friends, work colleagues and religious institutions) and QOL domain scores (physical, psychological, social relationship and environmental QOL).

**Table 2:** Correlation between respondents’ characteristics, source of social support and quality of life scores (*N* = 239).

Variables	1	2	3	4	5	6	7	8	9	10	11	12	13	14
(1) Age (years)	–													
(2) HIV Infection^a^	.09	–												
(3) Pleuritis with pleural effusion^a^	.01	.05	–											
(4) Duration on TB Treatment (days)	-.21**	-.03	-.15*	–										
(5) Number of Dependants	.54***	.23***	-.05	-.16*	–									
(6) Distance to TB centre (km)	.26***	.06	.23***	-.26***	.06	–								
(7) Physical QOL	-.25***	-.18**	-.17**	.09	-.14*	-.16*	–							
(8) Psychological QOL	-.12	-.22**	-.08	.09	-.07	-.14*	.80***	–						
(9) Social relationship QOL	.11	-.27***	-.13*	.12	-.04	-.13*	.68***	.74***	–					
(10) Environmental QOL	.14*	-.15*	-.07	.01	.11	-.02	.43***	.69***	.52***	–				
(11) Support from Family^a^	.11	-.08	.09	-.07	.03	.07	.05	.18**	.08	.35***	–			
(12) Support from Friends^a^	.06	.10	-.15*	.08	-.02	-.02	.18**	.14*	.12	-.03	-.59***	–		
(13) Support from Work colleagues^a^	-.18**	-.06	-.12	.01	-.09	-.10	-.08	-.14*	-.04	-.14*	-.34***	-.17**	–	
(14) Support from Religious Institutions^a^	-.08	.05	.16*	-.01	.06	.02	-.22**	-.31***	-.23***	-.34***	-.44***	-.22**	-.13*	–

*p < .05

**p < .01

***p < .001 (2-tailed) QOL = Quality of Life. TB = Tuberculosis. HIV = Human Immunodeficiency Virus.

^a^Dichotomous variable km = kilometres

## Discussion

The current study investigated the correlates of QOL among PLWTB within Greater Accra, Ghana. The study found a positive correlation among all four QOL domains. This finding suggests that higher levels of QOL in one QOL domain correspond with higher levels of QOL in the other three QOL domains [33, 34]. Thus, healthcare interventions that contribute to an improved Physical QOL for instance may also contribute to an improvement in the Psychological, Social and Environmental QOL of PLWTB and vice versa. Similarly, a deterioration in any of the QOL domains can correspond with a cascading deterioration in the other QOL domains of the PLWTB. This implies that healthcare programs or interventions for the management of TB should not only focus on curing TB disease but can consider all domains of the patients’ QOL [35].

It was also evident from this study that the characteristics of PLWTB (such as age, HIV infection, pleuritis with pleural effusion, number of dependants and distance to TB treatment centres) and their main source of social support (either family, friends, work colleagues or religious institution) correlated with their Physical, Psychological, Social relationship and Environmental QOL. Of particular interest was the finding that HIV infection corresponded with poorer Physical, Psychological, Social and Environmental Health QOL among the PLWTB. This finding is consistent with previous studies in Ethiopia, India, Nigeria and South Africa indicating that PLWTB-HIV coinfection simultaneously experience more deleterious effects of TB and HIV on their QOL when compared to their mono-infected (TB only or HIV only) counterparts [26, 33, 36–38]. The PLWTB-HIV coinfection suffer frequent or worse physical symptoms (such as weakness and physical pains) which may limit their ability to work or perform activities of daily living [39, 40]. Furthermore, the double stigma associated with TB-HIV coinfection can contribute to psychological problems (such as blue mood and depression) as well as social isolation and loss of employment [37, 41]. These challenges coupled with the significantly higher number of dependants among the PLWTB-HIV coinfection suggest that healthcare professionals may have to prioritise the support and care they provide to PLWTB-HIV coinfection who are at a higher risk of poor QOL. Graduating student nurses in the study setting have been found to lack the skills required to provide psychological and emotional support to PLWTB-HIV in their care [42]. The training of healthcare professionals could include content which equips them to promote the QOL of PLWTB.

Older age, having pleuritis with pleural effusion and a higher number of dependants individually corresponded with poorer Physical QOL among the PLWTB. The finding that age was associated with Physical QOL is comparable to previous studies in South Africa [24] and Nigeria [43] but contrary to studies in Ethiopia [17], Indonesia [44] and Jordan [45]. Older age may have corresponded with poorer Physical Health QOL because people experience degenerative changes in their body as they age thus may experience poorer physical QOL when infected with a chronic and debilitating infection like TB [46]. Additionally, the higher number of dependants associated with older age in this study also poses a potential problem of older PLWTB being overburdened by their dependants’ needs while self-managing TB themselves [40]. Considering the dissaving and negative socio-economic impact associated with TB, older PLWTB may require help in addressing not only their own health care needs but their dependants’ needs as well [39].

Longer distance away from the TB treatment centre correlated with poorer Physical, Psychological and Social relationship QOL among the PLWTB. Interestingly, older age corresponded with seeking treatment from further TB treatment centres despite its associated transportation cost. Sometimes, PLWTB prefer treatment centres that are located away from their communities as a measure to avoid stigmatization from community members or healthcare providers [47, 48]. However, seeking TB-related healthcare over long distances can be physically stressful for PLWTB and carries a risk of treatment non-adherence if the ease and cost of transportation become challenging over time [48, 49]. The findings reinforce the need for healthcare providers to take cognisance of the physical, psychological and social QOL well-being of PLWTB who prefer TB treatment centres situated away from their communities.

Pleuritis with pleural effusion among the PLWTB correlated with poorer Physical and Social relationship QOL as well as seeking treatment in further TB treatment centres. The diagnosis and treatment for pleuritis with pleural effusion involves thoracentesis or thoracocentesis which is an invasive medical procedure that may not be available in all TB treatment centres in Ghana [50]. Thoracentesis or thoracocentesis is the removal of accumulated fluid or air from the pleural space around the lungs for the purpose of diagnosing or treating pleuritis with pleural effusion [51]. Consequently, PLWTB-pleuritis with pleural effusion who require thoracentesis may have to travel over long distances for treatment in selected health facilities. Furthermore, the poorer Social relationship QOL among the sampled PLWTB-pleuritis with pleural effusion suggests they were not satisfied with their sex life, personal relationships and support received from friends. These inferences are reinforced by the observation in this study that PLWTB-pleuritis with pleural effusion received significantly less support from their friends despite having poorer Physical Health QOL. Fear of TB infection and inadequate knowledge among relations of PLWTB can contribute to diminished social support to PLWTB, necessitating further public health education [48, 52]. To mitigate the impact of TB on the QOL of PLWTB-pleuritis with pleural effusion, there is the need for care providers to discuss appropriate scheduling of follow-up visits, alternative transportation and sources of social support with PLWTB-pleuritis with pleural effusion.

Majority of the PLWTB in this study were newly diagnosed PLWTB in the intensive phase (first 2 months) of their treatment. The intensive phase can be physically and socially excruciating for PLWTB receiving TB treatment for the first time thus could negatively affect their QOL [53]. Previous studies emphasise the relevance of social support to the ability of PLWTB to cope with the impact of TB on their lives [23, 54, 55] and faith-based or religious institutions play a key role in providing this social support to PLWTB globally [22]. It was therefore remarkable to observe in this study that receiving support mainly from religious institutions corresponded with poorer QOL across all four domains (Physical, Psychological, Social relationship and Environmental QOL). Similarly, receiving support mainly from work colleagues correlated with poorer Psychological and Environmental QOL among the PLWTB. These related findings suggest that religious institutions and workplaces/colleagues may be supporting abandoned PLWTB with poorer QOL. The finding could also mean that religious institutions and work colleagues may not necessarily be the best sources of social support for PLWTB. This conclusion is strengthened by the findings that (1) receiving support primarily from the family corresponded with higher Psychological and Environmental Health QOL levels and (2) receiving support from friends correlated with higher Physical and Psychological Health QOL among PLWTB. Although contrary to findings in Pakistan [25], the observations in this study that family members’ support was associated with higher QOL was not surprising since, in most settings, the family plays a significant role in patients’ daily care and ability to cope with the challenges associated with living with TB [23, 56]. The family or friends of PLWTB may also volunteer as treatment supporters who encourage the PLWTB to adhere to their treatment regimen [57]. In line with these observations, the majority of the PLWTB in this study suggested support from family, children, friends and parents as possible ways of lessening the impact of TB on their QOL. Accordingly, healthcare professionals can consider strategies such as self-management support or counselling [58] in order to involve the families and friends of PLWTB in their care to improve their QOL.

## Conclusion

This study revealed that the physical, psychological, social relationship and environmental QOL domains of PLWTB were interrelated hence an improvement in one QOL domain corresponded with an improvement in the other three QOL domains. Furthermore, receiving social support from family or friends corresponded with better QOL among PLWTB. Inversely, receiving social support mainly from work colleagues or religious institutions correlated with poorer QOL among the PLWTB. Older age, HIV infection, pleuritis with pleural effusion as well as staying further away from the TB treatment centre corresponded with poorer QOL. The QOL of PLWTB should therefore be considered during their treatment. Future research can investigate interventions that improve the QOL of PLWTB.

### Study limitations and strengths

A limitation of this study is that it was conducted in a predominantly urban setting and thus may not represent the QOL of PLWTB in rural settings. Secondly, the study emphasised the correlational relationship among respondents’ characteristics, their main source of social support against TB and their QOL domains. These correlational relationships among the variables do not establish causality thus findings must be interpreted as such. Despite these limitations, the study can inform health providers’, investigators’ and educators’ practice, research and training related to the QOL of PLWTB.

## Supporting information

S1 File World Health Organization Quality of Life Brief version (WHOQOL-BREF) questionnaire. (PDF)
