# Supplementary material for: Correlates of quality of life among persons living with tuberculosis: A cross-sectional study
Source: PLoS One. 2022 Nov 4;17(11):e0277192. doi: 10.1371/journal.pone.0277192 (PMC9635747; doi:10.1371/journal.pone.0277192)
Supplement: S1 File — (PDF) [file pone.0277192.s001.pdf]

QUESTIONNAIRE ON THE IMPACT OF TUBERCULOSIS ON THE QUALITY OF LIFE OF PERSONS WITH TUBERCULOSIS IN GREATER ACCRA, GHANA

Questionnaire Number: ______________ Date: ___________________
Study Site: ________________________

SECTION ONE: RESPONDENT'S CLINICAL INFORMATION

1. Category of Tuberculosis
   [ ] Pulmonary
   [ ] Extrapulmonary ***(Specify affected organ)***………………………………………

2. Category of Case
   [ ] New case
   [ ] Re-treatment

3. Phase of Treatment
   [ ] Intensive Phase
   [ ] Continuation Phase

4. Duration on Tuberculosis Treatment **(*in Days*)** …………………………………

5. Co-morbidity status ***(Tick as many as apply)***
   [ ] HIV
   [ ] Pleural Effusion
   [ ] Diabetes
   [ ] Others ***(specify)***………………………………………………………………..

SECTION TWO: RESPONDENT'S SOCIO-DEMOGRAPHIC CHARACTERISTICS

6. Age ***(in years)***….………………..….

7. Sex
   [ ] Female [ ] Male

8. Marital Status
   [ ] Single [ ] Married [ ] Separated [ ] Others ***(specify)***…………..……

9. Highest Level of education
   [ ] No formal education
   [ ] Primary (Primary School, Junior High/Secondary School)
   [ ] Secondary(Senior High/Secondary School)
   [ ] Tertiary (College/Polytechnic/University)
   [ ] Other…………………………

10. Distance between your residence and TB treatment center ***(in kilometers)*** ……………

11. Occupation
    [ ] Unemployed
    [ ] Student
    [ ] Others………………………………………………………………..

12. Number dependents ***(specify)***…………………………………………….

SECTION THREE: IMPACT OF TUBERCULOSIS

Please read the following questions and choose your appropriate answer.

		Very poor	Poor	Neither poor nor good	Good	Very good
15.	How would you rate your quality of life?	1	2	3	4	5

		Very dissatisfied	Dissatisfied	Neither satisfied nor dissatisfied	Satisfied	Very satisfied
16.	How satisfied are you with your health?	1	2	3	4	5

The following questions ask about **how much** you have experienced certain things in the last two weeks.

		Not at all	A little	A moderate amount	Very much	An extreme amount
17.	To what extent do you feel that physical pain prevents you from doing what you need to do?	5	4	3	2	1
18.	How much do you need any medical treatment to function in your daily life?	5	4	3	2	1
19.	How much do you enjoy life?	1	2	3	4	5
20.	To what extent do you feel your life to be meaningful?	1	2	3	4	5

		Not at all	A little	A moderate amount	Very much	Extremely
21.	How well are you able to concentrate?	1	2	3	4	5
22.	How safe do you feel in your daily life?	1	2	3	4	5
23.	How healthy is your physical environment?	1	2	3	4	5

The following questions ask about how completely you experience or were able to do certain things in the last two weeks.

		Not at all	A little	Moderately	Mostly	Completely
24.	Do you have enough energy for everyday life?	1	2	3	4	5
25.	Are you able to accept your bodily appearance?	1	2	3	4	5

26.	Have you enough money to meet your needs?	1	2	3	4	5
27.	How available to you is the information that you need in your day-to-day life?	1	2	3	4	5
28.	To what extent do you have the opportunity for leisure activities?	1	2	3	4	5

		Very poor	Poor	Neither poor nor good	Good	Very good
29.	How well are you able to get around?	1	2	3	4	5

		Very dissatisfied	Dissatisfied	Neither satisfied nor dissatisfied	Satisfied	Very satisfied
30.	How satisfied are you with your sleep?	1	2	3	4	5
31.	How satisfied are you with your ability to perform your daily living activities?	1	2	3	4	5
32.	How satisfied are you with your capacity for work?	1	2	3	4	5
33.	How satisfied are you with yourself?	1	2	3	4	5

34.	How satisfied are you with your personal relationships?	1	2	3	4	5
35.	How satisfied are you with your sex life?	1	2	3	4	5
36.	How satisfied are you with the support you get from your friends?	1	2	3	4	5
37.	How satisfied are you with the conditions of your living place?	1	2	3	4	5
38.	How satisfied are you with your access to health services?	1	2	3	4	5
39.	How satisfied are you with your transport?	1	2	3	4	5

The following question refers to how often you have felt or experienced certain things in the last two weeks.

		Never	Seldom	Quite often	Very often	Always
40.	How often do you have negative feelings such as blue mood, despair, anxiety, depression?	5	4	3	2	1

		Not at all	Not much	Moderately	A great deal	Completely
41.	Do you get the kind of support from others that you need?	5	4	3	2	1

42. Which of the following offers you the most support in dealing with the impact of Tuberculosis?
(Tick only one)

[ ] Family
[ ] Friends
[ ] Work colleagues
[ ] Religious Institution (Church/ Mosque)
[ ] Others

(specify)……………………………………………………………………………..

Reproduced with permission from World Health Organization (WHO), The World Health Organization quality of life (WHOQOL) - BREF. 2012; https://apps.who.int/iris/handle/10665/77773*, Geneva. accessed (19/10/2022). WHO does not endorse any specific companies, products or services. (ID 390840)*
